# Current Recommendations, Antibiotic Resistance, and Emerging Treatments for Gram-Positive Ocular Infections in the United States

**DOI:** 10.3390/antibiotics15070710

**Published:** 2026-07-22

**Authors:** Inès Fenniri, Camille André

**Affiliations:** 1 Department of Ophthalmology, Massachusetts Eye and Ear, Harvard Medical School, 243 Charles Street, Boston, MA 02114, USA; ifenniri@meei.harvard.edu; 2 Infectious Disease Institute, Massachusetts Eye and Ear, Harvard Medical School, Boston, MA 02114, USA; 3 Department of Ophthalmology, Boston Children’s Hospital, Harvard Medical School, 300 Longwood Avenue, Boston, MA 02115, USA; 4 Department of Ophthalmology, Hôpital Edouard Herriot, Hospices Civils de Lyon, Université Claude Bernard Lyon 1, 5 Place d’Arsonval, 69003 Lyon, France

**Keywords:** Gram-positive ocular infection, antimicrobial resistance, MRSA, vancomycin-alternative therapy, endophthalmitis, antimicrobial stewardship, ocular pharmacokinetics

## Abstract

Severe bacterial keratitis and endophthalmitis are leading causes of corneal scarring, enucleation, and permanent vision loss, and are increasingly complicated by multidrug-resistant Gram-positive ocular pathogens. In the United States, according to the ARMOR surveillance program (2009–2020), methicillin resistance reached 45.9% of intraocular *Staphylococcus aureus* and 47.9% of coagulase-negative staphylococci (CoNS), with multidrug resistance exceeding 70% among methicillin-resistant intraocular isolates. While new antibiotics have been approved for systemic infections, vancomycin remains the standard of care for severe Gram-positive ocular infections. However, its ophthalmic use is constrained by limited ocular surface tolerability, physical incompatibility with intravitreal ceftazidime, and an association with hemorrhagic occlusive retinal vasculitis. To address this gap and further stimulate the development of next-generation targeted therapies in ophthalmology, this review focuses on current recommendations and new vancomycin alternative treatments for Gram-positive ocular infections in the antibiotic resistance era. This review details the current antimicrobial recommendations for the most frequent ocular infections, followed by the epidemiology and molecular mechanisms of resistance in *S. aureus*, CoNS, and *S. pneumoniae*. Finally, this review discusses new drugs that represent promising alternatives to vancomycin in ophthalmology.

## 1. Introduction

Ocular infectious diseases rank among the leading causes of ocular morbidity and vision loss worldwide, with bacteria being the most common etiologic agents [1,2,3,4]. Bacteria are associated with distinct types of eye infections involving different anatomical compartments, including infections of the tissues surrounding the eye such as orbital cellulitis [5,6,7], ocular surface infections such as conjunctivitis [8,9] or keratitis [10,11], and intraocular infections such as endophthalmitis [12,13]. Their management differs by tissue kinetics and route of drug delivery [14].

Gram-positive bacteria are the leading cause of these infections in the United States (U.S.). *Staphylococcus aureus*, coagulase-negative staphylococci (CoNS), and *Streptococcus pneumoniae* are consistently identified as the principal ocular pathogens across keratitis, endophthalmitis, and conjunctivitis by the Ocular Tracking Resistance in U.S. Today program (Ocular TRUST, 2005–2008) [15] and the multicenter Antibiotic Resistance Monitoring in Ocular micRoorganisms study (ARMOR, since 2009) [16]. ARMOR has documented persistent methicillin and multidrug resistance (MDR) in ocular *S. aureus* and CoNS isolates, with the highest resistance burden in intraocular isolates. Among these isolates (aqueous and vitreous) collected from 2009 to 2020, 45.9% of *S. aureus* and 47.9% of CoNS were methicillin-resistant, with MDR exceeding 70% in methicillin-resistant strains [17]. Antimicrobial resistance in ophthalmology is particularly worrisome as treatment failures due to resistance can result in permanent tissue damage and severe consequences, such as blindness or loss of the affected eye [18,19,20,21]. Beyond ophthalmology, bacterial antimicrobial resistance was associated with an estimated 1.27 million deaths globally in 2019 [22]. Interpretation of antibiograms from ocular isolates remains limited by the absence of ocular-specific breakpoints, as categorization still relies on systemic Clinical and Laboratory Standards Institute thresholds [23] developed for non-ocular infections treated with oral or intravenous antibiotics [16].

Fortified topical vancomycin has been used for decades in ophthalmology and continues to be the drug of choice recommended by the American Academy of Ophthalmology Bacterial Keratitis Preferred Practice Pattern for treatment of culture-documented cases of keratitis caused by MDR Gram-positive bacteria. It is also commonly used as part of empirical combination therapy in cases where no causative organism has been identified [24]. Although ocular staphylococci remain broadly susceptible to vancomycin [17], its ophthalmic use remains challenged by ocular surface tolerability, intravitreal incompatibility with ceftazidime, risk of hemorrhagic occlusive retinal vasculitis, and slow bactericidal activity against MDR staphylococcal strains [25,26,27,28,29,30].

To address this gap, this review focuses on current recommendations and new vancomycin alternative treatments for Gram-positive ocular infections in the antibiotic resistance era. First, this review summarizes the current antimicrobial recommendations for the most frequent ocular infections, followed by the epidemiology and molecular mechanisms of resistance in *S. aureus*, CoNS, and *S. pneumoniae*. The final section discusses new vancomycin alternative treatments, comparing their compartment-specific pharmacokinetics and ocular tolerability.

## 2. Methods

This narrative review was informed by a literature search of PubMed/MEDLINE from database inception through 13 July 2026. Search concepts combined terms related to ocular infection (ocular infection, eye infection, conjunctivitis, ocular surface infection, keratitis, endophthalmitis, intraocular infection, orbital cellulitis, periocular infection, lacrimal infection and dacryocystitis), Gram-positive pathogens (*Staphylococcus aureus*, coagulase-negative staphylococci, *Streptococcus pneumoniae*, *Enterococcus* spp., MRSA, MRCoNS, and VRE), antimicrobial resistance, and the established or emerging antimicrobial agents discussed in this review (vancomycin, linezolid, tedizolid, daptomycin, dalbavancin, oritavancin, telavancin, ceftaroline, delafloxacin, synthetic retinoids, CD437, CD1530, iboxamycin, cresomycin, bacteriophages, and endolysins).

## 3. Current Recommendations for Antibiotic Therapy

### 3.1. Orbital Cellulitis

Initial antibiotic therapy for orbital cellulitis is empirical, since reliable culture results are difficult to obtain in the absence of surgical intervention. For uncomplicated cases, treatment generally lasts 2–3 weeks. For severe ethmoid sinusitis or sinus bony destruction, a longer course of at least 4 weeks is recommended [31]. Vancomycin is the first-line agent for methicillin-resistant *S. aureus* (MRSA) coverage in orbital cellulitis. Intravenous antibiotic therapies for empiric treatment in patients presenting with orbital cellulitis are summarized in Table 1 [14].

In patients with a penicillin and/or cephalosporin allergy, treatment with a combination of vancomycin plus ciprofloxacin (400 mg IV twice per day) or levofloxacin (500 to 750 mg IV per day) is recommended.

For uncomplicated orbital cellulitis with a good response to IV antibiotics, it is possible to switch to oral therapy including clindamycin or trimethoprim-sulfamethoxazole plus amoxicillin–clavulanate or cefpodoxime or cefdinir.

### 3.2. Lacrimal System

Non-severe lacrimal infections can be treated with topical antibiotics such as azithromycin 1% for infections caused by Gram-positive organisms, or tobramycin 0.3% for Gram-negative organisms. However, for acute and chronic dacryocystitis, systemic antibiotics are needed and should cover the microorganism identified by culture. In MRSA dacryocystitis, intravenous vancomycin is the first-line agent [34]. In dacryocystitis caused by other Gram-positive organisms, amoxicillin–clavulanate or gentamicin are first-line agents [34]. If anaerobes are suspected, metronidazole or clindamycin are used.

### 3.3. Conjunctivitis

Because bacterial conjunctivitis is usually self-limited, a 5- to 7-day course of a broad-spectrum topical antibiotic is generally effective [35]. For non-severe conjunctivitis, the choice of antibiotic is usually empiric. In cases of moderate to severe conjunctivitis, the choice of antibiotic is guided by the results of laboratory tests. Antibiotic therapies for treatment of patients presenting with conjunctivitis are summarized in Table 2.

### 3.4. Keratitis

Topical antibiotic eye drops are the preferred method to treat most cases of keratitis (Table 3) [24]. For central or severe keratitis (e.g., deep stromal involvement or an infiltrate larger than 2 mm with extensive suppuration), an intensive loading regimen, for example, every 5–15 min initially, followed by hourly administration, is recommended. Subconjunctival antibiotics may be helpful where there is imminent scleral spread or perforation, or when adherence to the topical regimen is uncertain. Systemic antibiotics are rarely needed, but they may be considered in severe cases where the infectious process has extended to adjacent tissues (e.g., the sclera) or when there is impending or frank perforation of the cornea. Systemic therapy is necessary in cases of gonococcal keratitis.

Fluoroquinolones are widely used to treat bacterial keratitis. Ciprofloxacin 0.3%, ofloxacin 0.3%, and levofloxacin 1.5% have been approved by the Food and Drug Administration (FDA) for the treatment of bacterial keratitis, but the fourth-generation 8-methoxy fluoroquinolones (gatifloxacin and moxifloxacin) are not yet FDA-approved for the treatment of bacterial keratitis, although they are widely used in clinical practice [24]. Gatifloxacin and moxifloxacin have been reported to have better coverage of Gram-positive pathogens than earlier generation fluoroquinolones in in vitro studies [37]. Moreover, in studies including some randomized controlled trials, both moxifloxacin and gatifloxacin performed at least as well as standard therapy, fortified cefazolin/tobramycin combination therapy, and potentially better than an earlier-generation fluoroquinolone, ciprofloxacin [38,39,40,41,42,43]. Besifloxacin 0.6% is a topical fluoroquinolone that was approved by the FDA in 2009 for bacterial conjunctivitis, and its activity against ocular bacterial pathogens is similar to that of the fourth-generation agents [44]. In vivo rabbit studies have shown potential utility in the management of acute bacterial keratitis [45,46]. Clinical use of besifloxacin in bacterial keratitis has also been reported [47].

Fortified topical antibiotic combinations may be considered for large and/or vision-threatening corneal ulcers, particularly when a hypopyon is present, and for eyes unresponsive to initial treatment [24]. In contact lens wearers, *Pseudomonas aeruginosa* is the predominant bacterial pathogen, making empirical antipseudomonal coverage indispensable [10,48].

### 3.5. Endophthalmitis

The intravitreal route of drug administration is the recommended route to treat endophthalmitis as it helps in achieving high concentrations of the drug in the vitreous cavity without associated systemic side effects. Initially, broad-spectrum antibiotics are used empirically: IVT vancomycin 1 mg/0.1 mL plus either ceftazidime 2.25 mg/0.1 mL or amikacin 0.4 mg/0.1 mL. Response should be reassessed after approximately 48 h based on visual acuity, pain, hypopyon, vitreous inflammation, and microbiological results. Failure to stabilize or improve may prompt repeat microbiological sampling, repeat intravitreal therapy, and/or pars plana vitrectomy [49,50,51,52]. Repeated amikacin injections are avoided because of retinal toxicity [53]. Standard intravitreal antibiotics are summarized in Table 4 [50,52,54,55,56].

### 3.6. Optimizing Current Care

In parallel with the development of vancomycin-sparing therapies, established diagnostic and therapeutic pathways should be optimized. In bacterial keratitis, corneal scraping and culture are particularly indicated for large, central, deep stromal, atypical, or treatment-refractory ulcers [24]. In suspected acute bacterial endophthalmitis, aqueous and/or vitreous specimens should be obtained whenever feasible before intravitreal antimicrobial administration; however, microbiological sampling should not delay prompt treatment [52]. Because culture-based diagnostic methods are often slow and lack sensitivity [57], novel molecular diagnostic approaches are increasingly being implemented in ophthalmology to enable more rapid and accurate identification of ocular pathogens and are the subject of dedicated reviews [57,58].

Treatment success also depends on accurate drug preparation, delivery, and follow-up. Fortified topical and intravitreal preparations should comply with sterile-compounding standards, and their concentration, vehicle, storage conditions, and beyond-use date should be based on formulation-specific stability and sterility data [59,60]. Vancomycin and ceftazidime should be prepared and administered separately because of their physical incompatibility [26,27]. Intensive topical regimens require clear dosing instructions, assessment of the patient’s ability to adhere to treatment, and close early follow-up.

Once microbiological results become available, therapy should be de-escalated or adjusted to the most appropriate active agent and route of administration [24,55] to limit unnecessary exposure to broad-spectrum antibiotics [55].

## 4. Epidemiology and Mechanisms of Resistance in Gram-Positive Bacteria Causing Eye Infections

### 4.1. Phenotypic Resistance

The Centers for Disease Control and Prevention (CDC) characterizes antibiotic resistance as a leading public health threat and a priority of global significance [61]. Antibiotic resistance among bacteria is an ongoing concern in all fields of medicine [22], including ophthalmology [16,62,63], where treatment failures can result in vision loss or enucleation [18,19,20,21].

The Ocular Tracking Resistance in U.S. Today (Ocular TRUST) study was the first nationwide surveillance program to track in vitro resistance specifically for bacterial isolates from ocular tissue sources. Ocular TRUST was conducted in the U.S. for 4 years (2005–2008) [15]. During the study time frame, the data indicated levels of methicillin resistance among *S. aureus* and CoNS isolates ranging from 17% to 54% and from 57% to 62%, respectively, as well as multidrug resistance (MDR) to other antibiotic classes [15].

The Antibiotic Resistance Monitoring in Ocular micRoorganisms (ARMOR) surveillance study, initiated in 2009, is the only U.S. multicenter and nationwide surveillance program specific to common ocular bacterial pathogens. It evaluates clinically relevant isolates of *S. aureus*, CoNS, *S. pneumoniae*, *P. aeruginosa*, and *H. influenzae* sourced from any ocular tissue as part of routine medical care. Ocular isolates are obtained from community and university hospitals, ocular centers, and reference laboratories across the U.S. Cumulatively over its first 10 years (2009–2018), the ARMOR study analyzed the in vitro antibiotic susceptibility profiles of >6000 ocular isolates from throughout the U.S. [16]. Antibiotic susceptibility profiles were determined using CLSI-approved interpretive breakpoint criteria [23], even though CLSI breakpoints used for susceptibility determinations are based on pharmacokinetics and pharmacodynamics of antibiotics when administered systemically. As there are no specific ocular breakpoints for susceptibility, systemic breakpoints are currently all that are available for assessing in vitro susceptibility, even though topical ocular administration is the major route used to treat ocular infections. Topical ocular administration produces much higher initial concentrations than systemic administration, but tear dilution and nasolacrimal drainage rapidly reduce ocular surface exposure. The clinical relevance of in vitro susceptibility data for ocular infections is unclear.

#### 4.1.1. *Staphylococcus aureus*

Methicillin resistance in ocular *S. aureus* isolates ranges from 24% to 55% in the U.S. [2,16,64,65,66]. In Europe, prevalence of methicillin resistance in ocular *S. aureus* is much lower (ranging from 3% to 33%) [67,68]. This reflects the general trends observed in antimicrobial susceptibilities among *S. aureus* from the SENTRY antimicrobial surveillance program over a 20-year period, showing a higher prevalence of MRSA in North America (47.0%) than in Europe (26.8%) [69]. In the ARMOR study (2009–2018), 34.9% of *S. aureus* isolates were resistant to oxacillin/methicillin. MRSA rates tended to be higher among isolates from endophthalmitis (46.9%) and keratitis (34.3%) compared to conjunctivitis (28.9%) [16]. Resistance to ciprofloxacin was observed in 33.5% and to azithromycin in 59.7% of *S. aureus* isolates [16].

Compared with methicillin-susceptible *S. aureus* (MSSA) isolates, resistance to other antibiotics was more prevalent among MRSA isolates, with resistance greater than 60% for fluoroquinolones (excluding besifloxacin, for which no CLSI systemic breakpoint is available because it is formulated only for ocular use) and 92.9% for azithromycin. The rate of multidrug resistance among all *S. aureus* isolates was 30.1%, whereas the rate among MRSA isolates was 75.4% [16]. An analysis of 1601 isolates from the Massachusetts Eye and Ear (2014–2021) found that 60% of MRSA and 76.1% of methicillin-resistant CoNS (MRCoNS) were MDR [66]. Further genomic analysis revealed that two MRSA lineages, CC5 and CC8, predominate in the U.S., including in ophthalmology [65,66,70].

The later generation fluoroquinolones (besifloxacin, gatifloxacin, and moxifloxacin) had lower MIC_90_ values overall than the earlier ones (ciprofloxacin, levofloxacin, and ofloxacin). Resistance to fluoroquinolones also varied by tissue source in the ARMOR study, with higher rates in endophthalmitis (42.9%) and keratitis (35.7%) compared to conjunctivitis (28.4%).

ARMOR and other studies consistently indicated high susceptibility of *S. aureus* to tetracycline (ARMOR, 93.9%; others, 78.0–84.0%), aminoglycosides (ARMOR, 84.4%; others, 75.0–95.0%), trimethoprim (ARMOR, 95.7%; others, 95.7%), and vancomycin (ARMOR, 100%; others, 99–100%) [2,16,62,64,71,72,73,74,75,76,77].

#### 4.1.2. Coagulase-Negative Staphylococci (CoNS)

In the ARMOR study, 49.3% of CoNS isolates were resistant to oxacillin/methicillin. Resistance to ciprofloxacin was observed in 32.2% and to azithromycin in 61.3% of CoNS isolates. Compared with methicillin-susceptible CoNS isolates, resistance to other antibiotics was more prevalent among MRCoNS isolates, with resistance greater than 40% for fluoroquinolones and 78.6% for azithromycin [16]. The rate of multidrug resistance among all CoNS isolates was 41.2%, whereas the rate among MRCoNS isolates was 73.7% [16].

In the ARMOR study, resistance to fluoroquinolones appeared to be impacted by tissue source, with endophthalmitis isolates (52.2%) showing higher resistance to these antibiotics compared to keratitis (36.1%) or conjunctivitis (29.2%) isolates [16,62]. Overall, susceptibility was high to tetracycline (87.6%), aminoglycosides (82.5%), and vancomycin (100%) [16]. Clinically, these resistance rates indicate that empirical β-lactams and fluoroquinolones are unlikely to provide optimal antimicrobial coverage against CoNS, especially MRCoNS, which are predominantly multidrug-resistant and that fluoroquinolone monotherapy should be avoided for intraocular MRCoNS infection. Intravitreal vancomycin remains the empirical Gram-positive agent of choice for MRCoNS endophthalmitis [16].

#### 4.1.3. *Streptococcus pneumoniae*

Data on ocular *S. pneumoniae* isolates suggest a high degree of susceptibility to fluoroquinolones and tetracycline, with resistance rates commonly below 10% [16,78]. In the ARMOR study, 67.8% of *S. pneumoniae* isolates were susceptible to penicillin. Resistance to macrolides is more common among ocular *S. pneumoniae*, with 35.9% of isolates collected by ARMOR being resistant to one or more molecules within this class.

Resistance of *S. pneumoniae* to β-lactams and macrolides is a major concern worldwide. The prevalence of penicillin-non-susceptible *S. pneumoniae* exceeds 20% among adults and 40% among children in the U.S. and Europe [79,80,81]. *S. pneumoniae* macrolide resistance in the U.S. and in Europe is relatively common, with overall rates usually ranging from 20% to 40%, with even higher rates among respiratory isolates [79,80,81,82,83]. These phenotypic resistance patterns reflect the acquisition and selection of defined molecular mechanisms in *S. aureus*, CoNS, and *S. pneumoniae*. 

### 4.2. International Context

The most comprehensive synthesis of antimicrobial resistance in ophthalmology to date is a 2026 systematic review and meta-analysis of 340 reports from 47 countries, encompassing 129,388 culture-positive episodes of bacterial keratitis [84]. Consistent with U.S. ARMOR findings, vancomycin susceptibility among Gram-positive bacterial keratitis isolates was 100.0%. The overall prevalence of MRSA was 20% [84], although considerable geographic variation was observed [84,85]. European series have generally reported lower methicillin-resistance burdens than U.S. surveillance [67,68]; however, one European ocular MRSA series reported resistance exceeding 60% to azithromycin, ciprofloxacin, and fusidic acid [86]. In East Asia, MRSA accounted for 52.8% of culture-proven ocular *S. aureus* infections over a 10-year period in Taiwan [87]. In a Chinese pediatric cohort, 71.1% of ocular MRSA isolates were multidrug resistant [88]. The following section summarizes the principal resistance determinants relevant to ophthalmic practice, by antibiotic class.

### 4.3. Mechanisms of Resistance

#### 4.3.1. Penicillin and Methicillin Resistance

β-lactam antibiotics inhibit bacterial cell-wall biosynthesis. Peptidoglycan is the main structural component of the cell wall, and it consists of glycan strands made of repeating N-acetylglucosamine and N-acetylmuramic acid disaccharides linked by peptide cross-links between N-acetylmuramic acid moieties on adjacent strands. Key steps in peptidoglycan biosynthesis include transglycosylation and transpeptidation. N-acetylglucosamine and N-acetylmuramic acid disaccharides are attached via a β-1,4-glycosidic bond by a transglycosylation reaction [89]. The newly incorporated repeating unit is cross-linked by a transpeptidation reaction to a stem peptide in an adjacent peptidoglycan strand. Both transglycosylation and transpeptidation are carried out by penicillin-binding proteins (PBPs); the latter reaction is the specific target of β-lactams [89]. β-lactams inhibit the transpeptidation step of cell-wall biosynthesis by acting as substrate analogs of the D-Ala D-Ala peptidoglycan side chain upon which PBPs act [90].

In *S. pneumoniae* isolates, β-lactam resistance is primarily driven by alterations in the transpeptidase domains of PBPs that reduce antibiotic-binding affinity. Alterations to the properties of PBPs are brought about by the transfer of portions of the genes encoding the PBPs from other streptococcal species, resulting in mosaic genes [91].

With the widespread use of penicillin in the 1950s, penicillin-resistant *S. aureus* emerged and spread globally [92]. These strains produced a penicillinase encoded by *blaZ*, that hydrolyzes the β-lactam ring of penicillin, essential for its antimicrobial activity [93].

The β-lactamase-resistant penicillin derivative, methicillin, was introduced in 1961, but MRSA strains appeared shortly thereafter [94]. Resistance was not due to β-lactamase production but due to the expression of an additional penicillin-binding protein, PBP2a, which has extremely low affinity for most β-lactam antibiotics [95]. In the presence of β-lactam antibiotics that inhibit the function of the four native *S. aureus* PBPs (PBP1, PBP2, PBP3 and PBP4), PBP2a can take over the transpeptidase function of peptidoglycan biosynthesis. PBP2a is primarily encoded by the gene *mecA* [96], which is located within a mobile genetic element named the staphylococcal cassette chromosome (SCC*mec*) [97]. SCC*mec* elements are highly diverse, with 15 types (I to XV) recognized to date [98,99,100,101,102,103]. Despite their diversity in size (from ∼21 kb to 67 kb) and gene content, they all share important defining characteristics used in the SCC*mec* classification [104]. Clinically, because PBP2a has low affinity for essentially all β-lactams, a methicillin-resistant isolate should be regarded as resistant to the entire penicillin and cephalosporin class used in ophthalmology; this is the rationale for empirical vancomycin in suspected ocular MRSA infection [89]. The fifth-generation cephalosporins (e.g., ceftaroline) are an exception, retaining anti-MRSA activity through high-affinity binding to PBP2a, but they lack an ophthalmic formulation (Section 5.8).

#### 4.3.2. Vancomycin Resistance

Vancomycin has been the first-line agent for invasive MRSA infections in hospitalized patients since the 1960s and remains the standard of care for severe ocular MRSA disease [24].

After many years of use, vancomycin intermediate *S. aureus* (VISA—showing in vitro vancomycin MIC values from 4 to 8 μg/mL) [105] appeared in Japan in 1997 [106], and since then, it has been identified worldwide. The VISA phenotype results from mutations acquired during antibiotic therapy and has been associated with treatment failures [106,107,108]. Several mutated genes have been identified in the VISA phenotype [109,110,111], mainly involving the cell-wall synthesis and resulting in increased wall thickness and altered architecture [112]. The most frequently mutated determinants identified in 22 clinical VISA isolates from the collection of the CDC and the New York City Department of Health and Mental Hygiene were the *walKR*, *vraSR*, and *rpoB* genes [109].

Heterogeneous VISA (hVISA) show MIC values in the susceptible range (≤ 2 μg/mL), but they contain a subpopulation that expresses a resistant phenotype [113]. This hVISA phenotype has direct consequences in ophthalmology: routine antimicrobial susceptibility reports may classify such isolates as vancomycin-susceptible, while empirical fortified topical or intravitreal vancomycin may demonstrate reduced clinical efficacy.

Vancomycin-resistant *S. aureus* (VRSA), defined by vancomycin MIC values ≥ 16 μg/mL [114], was first reported in the United States in 2002 [115]. Resistance is mediated by acquisition of the *vanA* operon from *Enterococcus faecalis* on the transposon Tn1546 [114]. *VanA* produces a dipeptide D-alanyl D-lactate, which has a lower affinity for vancomycin [116]. VRSA remains exceptionally rare, likely because *vanA* acquisition imposes a substantial fitness cost on *S. aureus* [117]. Nonetheless, sporadic case reports of ocular VRSA disease have been documented, including a case of recurrent VISA/VRSA interstitial keratitis managed with topical and systemic bacteriophage therapy after failure of conventional therapy [118].

#### 4.3.3. Fluoroquinolone Resistance

Quinolone Resistance-Determining Region (QRDR) mutations

Bacterial resistance to fluoroquinolones (FQs) arises primarily from mutations in bacterial topoisomerase II and topoisomerase IV genes [119,120]. Both enzymes are composed of two types of subunits, called GyrA and GyrB in topoisomerase II, and ParC and ParE in topoisomerase IV [121]. Single amino acid changes in either topoisomerase II or topoisomerase IV can cause quinolone resistance. These resistance mutations have most commonly been localized to the amino-terminal domains of GyrA (residues 67 to 106 for *Escherichia coli* numbering) or Par*C* (residues 63 to 102) [119]. These domains have been termed the “quinolone resistance determining region” (QRDR) of GyrA and ParC [122]. Resistance mutations that alter the structure of the GyrB and ParE subunits are less common than those altering the structure of GyrA and ParC subunits [123,124,125].

The magnitude of resistance caused by single amino acid changes in the subunits of gyrase or topoisomerase IV varies by bacterial species and by quinolone [65,125,126].

In the 10-year ARMOR dataset, besifloxacin MIC_90_ values were at least 4-fold lower than moxifloxacin and gatifloxacin and at least 16-fold lower than ciprofloxacin and levofloxacin for Gram-positive bacteria including *S. aureus*, CoNS, and *S. pneumoniae* [16]. That besifloxacin had greater potency against FQ-resistant strains suggests that besifloxacin is less affected by topoisomerase mutations compared with older FQs. The study conducted by Sanfilippo et al. demonstrated that, as the number of mutations in genes encoding topoisomerase II and topoisomerase IV increases, MIC values increase for all tested FQs (besifloxacin, moxifloxacin, gatifloxacin, ciprofloxacin, and levofloxacin); the magnitude of this increase for besifloxacin, however, was the smallest (128-fold between susceptible and the most resistant strains) compared to all other FQs (1024- to 2048-fold) [127]. These resistance patterns do not support the use of empirical fluoroquinolone monotherapy for severe ocular infection.

Efflux pumps

Because topoisomerase II and topoisomerase IV are cytoplasmic enzymes, FQs must traverse the bacterial envelope to reach their targets, and efflux pumps that result in reductions in cytoplasmic drug concentrations can confer resistance. Low-level resistance to FQs can occur by increased expression of these efflux pumps, usually due to mutations that increase transcription of the structural gene for the pump, either from effects on the gene promoter or from alterations in other regulatory proteins that affect pump expression.

In *S. aureus*, overexpression of each of the three efflux pumps, NorA [128,129], NorB [130], and NorC [131], has been shown to cause low-level resistance to FQs, with some variations in substrate profiles among the three pumps. All three pumps are members of the major facilitator superfamily (MFS). NorA expression confers resistance to hydrophilic quinolones, such as norfloxacin and ciprofloxacin, whereas NorB and NorC expression each confers resistance to hydrophilic quinolones and hydrophobic quinolones, such as moxifloxacin [129,130,131]. These pumps also have substrate profiles extending beyond quinolones, in keeping with the broad substrate profiles of many MFS transporters.

Other Gram-positive transporters affect fluoroquinolone susceptibility but have been less extensively characterized than the Nor pumps. In *S. aureus*, overexpression of MFS transporters MdeA [132], SdrM [133], QacB(III) [134], and LmrS [135] has also been shown to reduce susceptibility to FQs.

In *S. pneumoniae*, an MFS transporter called PmrA [136] as well as an ATP-binding-cassette (ABC) transporter called PatAB [137], also contribute to reduced susceptibility to FQs.

#### 4.3.4. Macrolide Resistance

Three main mechanisms of antimicrobial resistance to the macrolides have been identified in Gram-positive bacteria: (i) target site alteration by ribosomal methylation; (ii) antibiotic efflux and (iii) antibiotic inactivation [138,139].

Ribosomal methylation

Methylation of the ribosomal target remains the most widespread macrolide resistance mechanism [139]. The MLS_B_ phenotype results from the action of *erm* (erythromycin ribosome methylase) genes [140]. Three major *erm* genes are detected in Gram-positive bacteria: *erm(A)*, *erm(B)*, and *erm(C)* [140,141]. *erm(A)* and *erm(C)* predominate in staphylococci, whereas *erm(B)* predominates in streptococci and enterococci [140]. *erm*-encoded methyltransferases dimethylate the N6 position of A2058 in the 23S rRNA of the 50S ribosomal subunit [139]. Because macrolides, lincosamides, and streptogramins B share overlapping binding sites at A2058, methylation produces cross-resistance to the three classes (MLS_B_ phenotype).

Expression of MLS_B_ resistance can be constitutive or inducible [138]. Inducible expression relies on an inactive mRNA that is structurally activated only in the presence of a macrolide inducer [138]; constitutive expression bypasses this requirement [138]. Strains harboring an inducible *erm* gene are resistant to the inducers but susceptible to non-inducing macrolides. Inducible MLS_B_ strains are detected by the erythromycin–clindamycin disk approximation test (D-test) [142]. Constitutive expression produces full cross-resistance to MLS_B_ antibiotics.

In staphylococci, the resistance phenotypes conferred by inducible expression of both determinants (*erm(A)* and *erm(C)*) confer dissociated MLS_B_ resistance: strains are resistant to inducing 14- and 15-membered macrolides [138], while non-inducing 16-membered macrolides and streptogramins B remain active (Table 5). Inducible expression of *erm(B)* results in a large variety of phenotypes, including resistance to 14-, 15- and 16-membered ring macrolides with susceptibility or resistance to clindamycin (Table 5) [143]. Ocular surveillance reproduces these distributions: in a genomic analysis of Gram-positive keratitis isolates at Massachusetts Eye and Ear, macrolide resistance was driven by *erm* genes in *S. aureus*, *mphC* and *msrA* in coagulase-negative staphylococci, and *mefA* coupled to *msr*(*D*) in streptococci [65]. Clinically, *erm*-mediated macrolide resistance among ocular staphylococci limits the reliability of topical erythromycin and azithromycin against these organisms [16].

Antibiotic efflux

Macrolide efflux in Gram-positive bacteria is mediated by the ATP-binding cassette family (ABC) and the major facilitator superfamily (MFS).

In *Staphylococcus* spp., *msr(A)* encodes an ABC-type efflux protein [144]. The functional pump combines *msr(A)* with auxiliary genes, conferring resistance to 14- and 15-membered macrolides and streptogramins B (MS_B_ phenotype) (Table 5) [144]. MS_B_ resistance can be inducible, with retained clindamycin susceptibility [144].

In *Streptococcus* spp., *mef(A)* encodes a proton-motive-force-driven MFS efflux pump [143]. This pump confers the M phenotype (resistance limited to 14- and 15-membered macrolides). *mef(A)* is co-localized with *msr(D)* (ABC transporter) to form a two-component pump (Table 5) [145,146,147].

Antibiotic inactivation

*Staphylococcus* spp. also produce phosphotransferases encoded by the *mph(C)* gene, conferring resistance to erythromycin and other 14- and 15-membered macrolides (M phenotype) (Table 5) [148].

#### 4.3.5. Aminoglycoside Resistance

As *Streptococcus* spp. lack sufficient oxidative metabolism to drive uptake of aminoglycosides, they have intrinsic low-level resistance to aminoglycosides [149].

In *Staphylococcus* spp., aminoglycoside resistance mainly results from antibiotic inactivation by aminoglycoside-modifying enzymes [150,151].

Three classes of aminoglycoside-modifying enzymes—acetyltransferases (AAC), phosphotransferases (APH), and nucleotidyltransferases (ANT)—confer high-level resistance by covalent modification that prevents ribosomal binding. The standard nomenclature is a three-letter activity code, a parenthesized site of modification, and a Roman-numeral subclass [151].

Acetyltransferases

AACs acetylate amino groups; most have been characterized in Gram-negative bacteria, but AAC(6′)-I has been identified in *S. aureus* [152]. It confers resistance to amikacin and tobramycin.

Phosphotransferases

APHs are frequent in *Staphylococcus* spp. isolates, especially APH(3’)-IIIa [153]. APHs phosphorylate hydroxyl groups, introducing a negative charge that abolishes ribosomal A-site binding.

APH(3’)-IIIa confers resistance to kanamycin and neomycin. In addition, APH(2″)-Ie can be found in methicillin-resistant staphylococcal isolates, conferring resistance to kanamycin, amikacin, gentamicin and tobramycin [153].

Nucleotidyltransferases

ANTs catalyze the transfer of an AMP group from an ATP donor to a hydroxyl group in aminoglycosides. ANT(6) enzymes are widespread among Gram-positive bacteria and confer resistance to streptomycin [152]. ANT(9) enzymes, also found in Gram-positive bacteria, confer resistance to spectinomycin, whereas ANT(4)-I confers resistance to tobramycin and amikacin [152].

Clinically, aminoglycosides act synergistically with antibiotics targeting the cell wall: by inhibiting peptidoglycan synthesis, β-lactams and glycopeptides enhance aminoglycoside uptake, producing bactericidal synergy that underlies their combination use in severe Gram-positive infections. However, this synergistic activity is abolished by the enzyme-mediated resistance described above, emphasizing the importance of detecting aminoglycoside resistance genes [154].

## 5. Potential New Treatments in Ophthalmology

### 5.1. Limitations of Vancomycin as the Standard of Care in Ophthalmology

Vancomycin has remained the standard of care for severe Gram-positive ocular infections for more than three decades, although several limitations related to ocular toxicity and formulation challenges have been recognized.

Fortified topical vancomycin may be poorly tolerated at the ocular surface. In a retrospective interventional case series of seven patients with bacterial keratitis switched from fortified topical vancomycin to topical linezolid 0.2% because of vancomycin intolerance, the mean ocular surface score (punctate epithelial erosions) was significantly higher during vancomycin than during linezolid treatment (9.4 ± 1.6 vs. 5.9 ± 1.3; *p* = 0.027) [28].

Intraocular vancomycin has also been associated with hemorrhagic occlusive retinal vasculitis (HORV), a rare but potentially blinding syndrome following intracameral or intravitreal administration during cataract surgery [25]. Accordingly, the Food and Drug Administration states that vancomycin is not indicated for endophthalmitis prophylaxis [155]. This restriction targets prophylactic use in uninfected eyes and does not apply to treatment: intravitreal vancomycin with ceftazidime remains the standard empirical regimen for acute bacterial endophthalmitis, covering Gram-positive (including MRSA) and Gram-negative (including *P. aeruginosa*) organisms pending cultures [52]—though the two are physically incompatible and must be prepared and given separately [156].

Newer Gram-positive drugs have since been approved for systemic indications [17,62], and may represent promising therapeutic alternatives to vancomycin for the management of ocular infections (Table 6).

### 5.2. Oxazolidinones (Linezolid, Tedizolid)

Linezolid is currently the best-documented vancomycin-sparing regimen for documented Gram-positive intraocular infection [157,159,184]. As an oxazolidinone, it inhibits bacterial protein synthesis at the level of the 50S ribosomal subunit by preventing formation of the initiation complex, and is active against major MDR Gram-positive pathogens relevant to ophthalmology, including MRSA and vancomycin-resistant enterococci (VRE) [158,185]. Fiscella et al. demonstrated that two 600-mg oral doses of linezolid administered 12 h apart produced mean aqueous and vitreous concentrations of 6.6 µg/mL and 5.7 µg/mL [157]. A later study by George et al. further showed that a single 600-mg oral dose yielded mean concentrations of 3.32 µg/mL in aqueous humor and 2.98 µg/mL in vitreous humor [159]. Brockhaus et al. reviewed intravitreal penetration of systemically administered antibiotics and identified linezolid, together with meropenem and moxifloxacin, as the systemic agent with the best-documented intravitreal penetration in human eyes [184]. Intraocular linezolid concentrations after twice-daily exposure exceed the CLSI susceptibility breakpoint for linezolid against staphylococci (≤4 µg/mL) [23], supporting oral or intravenous linezolid 600 mg twice daily rather than single-dose regimens for adjunctive use in Gram-positive endophthalmitis [184].

Topical linezolid 0.2% has also been investigated in ophthalmology [24,28,162]. In the case series reported by Tu and Jain, three patients with culture-confirmed Gram-positive isolates (one VRE and two infectious crystalline keratitis cases caused by an uncharacterized *Staphylococcus* and a *Streptococcus mitis*), all showed clinical resolution, and the authors concluded that topical linezolid was substantially more comfortable and less immediately toxic to the ocular surface than topical vancomycin [162]. A separate retrospective interventional case series of seven patients similarly reported better ocular surface tolerability with topical linezolid 0.2% than with topical vancomycin in bacterial keratitis [28]. These limited but consistent data support considering linezolid 0.2% as an alternative option for Gram-positive ocular surface infection caused by isolates resistant to first-line regimens or with reduced vancomycin susceptibility, particularly in patients with poor tolerance to fortified vancomycin in Gram-positive keratitis [24,28,162].

The intravitreal linezolid literature is much scarcer [160,161,184]. In a rabbit *S. aureus* endophthalmitis model, intravitreal linezolid showed concentration-dependent killing across doses of 1, 10 and 30 mg per eye, sterilizing all infected eyes at the highest dose tested (30 mg) on day 5, with a vitreous half-life of approximately 2 h and no electroretinographic toxicity at the 30 mg dose under the reported experimental conditions [160]. To our knowledge, only one human case report has been identified describing a postoperative VRE (*Enterococcus faecium*) endophthalmitis managed with intravitreal linezolid 200 µg/0.1 mL, after failure of repeated intravitreal vancomycin, ceftazidime, and amphotericin B as well as systemic linezolid; the infection cleared within a week, although a residual exudative retinal detachment was observed [161]. Further intravitreal pharmacokinetic, in vivo efficacy studies and larger clinical case series are needed to define the dose, repeat-injection schedule, and indication of intravitreal linezolid to treat endophthalmitis [160,161,184].

In addition to myelosuppression that warrants hematologic monitoring, linezolid also has adverse effects of particular relevance to ophthalmologists. Peripheral and optic neuropathy have been reported predominantly with treatment beyond 28 days, with prompt ophthalmic evaluation recommended when visual symptoms occur [158].

Linezolid resistance can emerge through 23S rRNA mutations, particularly G2576T, and through transferable oxazolidinone resistance genes such as *cfr*, *optrA*, and *poxtA*, making stewardship particularly important if linezolid use expands in ocular infections [185].

Tedizolid is a second-generation oxazolidinone that retains activity against *cfr*-positive linezolid-resistant Gram-positive bacteria [186].

Tedizolid has been evaluated primarily for ocular formulation development; clinical ophthalmic use has not been reported to date [187,188]. Ocular studies using chitosan nanoparticles and positively charged nanocrystals showed improved transcorneal permeation and ocular bioavailability in preclinical models [187,188]. These data support the pharmaceutical feasibility of topical ocular tedizolid delivery; however, clinical efficacy studies in ocular infections remain lacking [187,188]. A recent case report of optic neuropathy after an 18-month course of systemic tedizolid suggests that optic neuropathy remains a safety concern with prolonged systemic use [189].

### 5.3. Daptomycin

Daptomycin remains investigational for routine ophthalmic use, and its intraocular application is constrained by a narrow therapeutic window and the potential for retinal toxicity (detailed below). Within safe dosing, it could nonetheless represent an alternative to vancomycin for severe Gram-positive ocular infection. Daptomycin exerts rapid concentration-dependent bactericidal activity through calcium-dependent membrane depolarization and retains activity against resistant Gram-positive pathogens relevant to ophthalmology, including MDR MRSA and MRCoNS [164,165]. Most ophthalmic studies of daptomycin have focused on intravitreal administration in in vivo endophthalmitis models. In the rabbit study by Comer et al. [163], intravitreal daptomycin showed a clear dose-response safety profile: 75 and 188 μg produced no clinical, electroretinographic, or histopathologic signs of retinal toxicity, whereas 375 and 750 μg produced dose-dependent cataract, electroretinographic suppression, and photoreceptor damage. In the same model, 200 μg of intravitreal daptomycin produced culture-negative vitreous in 1/7 eyes at 24 h and in 7/7 infected eyes at 48 h in experimental *S. epidermidis* endophthalmitis [163].

Subsequent studies reinforced this translational impact. In a rabbit uveitis model, intravitreal daptomycin displayed a vitreous half-life of approximately 25.7 h in healthy eyes and 34.6 h in inflamed eyes, suggesting sustained intraocular exposure after injection [166]. After frequent topical instillation (1% daptomycin, one drop every 15 min for 1 h), mean rabbit corneal and aqueous concentrations reached 3.9–4.1 µg/g and 1.7–1.9 µg/mL, whereas a single drop left aqueous levels undetectable [168]. In an experimental MRSA keratitis model, topical daptomycin significantly reduced the corneal bacterial load, comparable to fortified vancomycin [169]. Human data remain limited. In a single patient with MRSA bacteremia and endogenous endophthalmitis, Sheridan et al. measured intravitreal daptomycin penetration after intravenous administration (10 mg/kg): the vitreous concentration reached 12.43 µg/mL, approximately 28% of the serum level and about 24-fold the isolate MIC (0.5 µg/mL) [167]. Because no intraocular cultures were obtained and intravitreal vancomycin and amphotericin B were co-administered, the study documents intravitreal penetration and clinical outcome in combination.

Two human case reports have described intravitreal daptomycin (200 µg/0.1 mL) therapy. In the first, a patient with MRSA endocarditis and presumed bilateral endogenous endophthalmitis due to a vancomycin-intermediate isolate (vancomycin MIC 4–8 µg/mL) received intravitreal daptomycin alongside intravenous daptomycin; the vitreous cleared completely in both eyes, with 20/20 visual acuity at 2 months [170]. In the second, intravitreal daptomycin resolved a recalcitrant postoperative endophthalmitis that had not responded to intravitreal vancomycin, in which Gram stain showed Gram-positive cocci but the organism remained unidentified, with repeatedly negative cultures [171].

Larger clinical, in vivo pharmacokinetic and efficacy, and dose-optimization studies are needed to define repeat-injection guidelines. The therapeutic window is the principal limitation, as several studies showed dose-dependent retinal toxicity at higher exposures [163,164,166].

### 5.4. Lipoglycopeptides (Dalbavancin, Oritavancin, Telavancin)

Lipoglycopeptides are semi-synthetic glycopeptides engineered to improve antibacterial potency against Gram-positive organisms [172]. Dalbavancin primarily interferes with cell-wall synthesis by binding to the D-alanyl-D-alanine terminus of nascent peptidoglycan and thereby preventing cross-linking [173]. Telavancin combines inhibition of late-stage peptidoglycan synthesis with direct disruption of bacterial membrane barrier function [175]. Oritavancin has the broadest mechanistic profile of the three agents, with inhibition of both transglycosylation and transpeptidation, together with membrane depolarization and permeabilization [174]. Dalbavancin and oritavancin also have prolonged terminal half-lives, approximately 346 and 245 h, respectively, whereas telavancin has a much shorter half-life of about 8 h [173,174,175]. Although prolonged systemic exposure may be advantageous for infrequent dosing, its ocular implications remain unknown. If substantial intraocular retention occurs, local toxicity could be prolonged and difficult to reverse; dedicated ocular pharmacokinetic studies are therefore required before intraocular administration of these agents can be considered.

Because of their enhanced antimicrobial potency and bactericidal activity, these newer lipoglycopeptides may represent promising therapeutic candidates for ocular infections [172,173,174,175]. Our in vitro study of 223 ocular *Staphylococcus* spp. isolates showed substantially greater in vitro potency than vancomycin [172]. Dalbavancin showed the lowest MIC_90_ (0.06 μg/mL), whereas telavancin and oritavancin each had MIC_90_ values of 0.12 μg/mL, compared with 2 μg/mL for vancomycin. Telavancin, dalbavancin, and oritavancin were also active against multidrug-resistant MRSA isolates, with equivalent MIC_90_ values [172]. Time-kill experiments identified oritavancin as the most rapidly bactericidal member of the class, with bactericidal activity achieved within 1 h against MRSA. Rapid bactericidal kinetics are relevant to severe ocular infection, where the bacterial inoculum is high and the therapeutic window is short [12,21].

A second major advantage supporting topical development of this class is corneal epithelial tolerance [172]. At a concentration commonly used for topical preparations (25 mg/mL), vancomycin reduced human corneal epithelial cell viability by approximately 50% after 5 min of incubation, whereas telavancin did not and the effect was less pronounced with oritavancin and dalbavancin [172]. Although all four agents showed time- and concentration-dependent cytotoxicity at 25 mg/mL after 30–60 min, the lipoglycopeptide MIC_90_ values are 15- to 30-fold lower than those of vancomycin, which would project therapeutic topical concentrations well below the cytotoxicity threshold observed in human corneal epithelial cells.

A murine maximum-tolerated-dose study provides additional in vivo ocular tolerability results for topical telavancin. At 25 mg/mL, telavancin produced only mild conjunctival changes in one of four eyes, whereas fortified vancomycin at the same concentration caused ocular irritation in all treated eyes [176].

All currently FDA-approved products are intravenous systemic formulations: dalbavancin and oritavancin are approved for acute bacterial skin and skin structure infections caused by susceptible Gram-positive organisms, whereas telavancin is approved for complicated skin and skin structure infections and for hospital-acquired or ventilator-associated bacterial pneumonia due to susceptible Gram-positive pathogens [173,174,175].

None currently has an established ophthalmic formulation or a labeled ophthalmic indication [173,174,175]. Further ocular pharmacokinetic studies, in vivo keratitis and endophthalmitis models, and topical formulation development are needed before lipoglycopeptides can be validated for clinical use in ophthalmology.

### 5.5. Synthetic Retinoids (CD437, CD1530)

Synthetic retinoids—especially CD437 and CD1530—act through a membrane-targeted antibacterial mechanism and are active against MRSA [177,190]. In the study by Kim et al., both compounds showed rapid bactericidal activity, synergized with gentamicin, and displayed a low probability of resistance selection [190]. They also identified a second-generation analogue (Analog 2) with an improved cytotoxicity profile and demonstrated efficacy in a mouse model of chronic MRSA infection [190].

We tested these synthetic retinoids against 216 ocular staphylococcal isolates, including 56 MRSA and 42 CoNS, and showed that their activity was maintained across multidrug-resistant phenotypes [177]. CD437 showed MIC_50_/MIC_90_ values of 2/2 μg/mL for MSSA and MRSA and 1/2 μg/mL for CoNS, whereas CD1530 showed MIC_50_/MIC_90_ values of 2/4 μg/mL for *S. aureus* and 2/2 μg/mL for CoNS [177]. Against MRSA and coagulase-negative staphylococci, retinoid potency exceeded that of ofloxacin, levofloxacin, moxifloxacin, and azithromycin and was comparable to that of vancomycin [177]. A separate *E. faecalis* study further showed that CD437 inhibits biofilm formation, kills mature biofilm, and acts synergistically with gentamicin in a systemic infection model, confirming retention of activity against biofilm-associated Gram-positive disease beyond ocular infections [178].

Synthetic retinoids remain preclinical candidates, with an interesting mechanism of action distinct from current drugs. The next milestones are ophthalmic formulation, ocular safety, pharmacokinetics, and in vivo efficacy models [177,190].

### 5.6. Iboxamycin and Cresomycin

Iboxamycin and cresomycin are next-generation synthetic lincosamides that inhibit bacterial protein synthesis by binding to the 50S large ribosomal subunit [180,181]. They were engineered to overcome lincosamide resistance mediated by widespread mechanisms including 23S rRNA methylation by *erm* and *cfr* genes, which confer MLS_B_ (macrolides, lincosamides, streptogramins B) and PhLOPS_A_ (phenicols, lincosamides, oxazolidinones, pleuromutilins, streptogramins A) resistance phenotypes, respectively.

Their activity against MLS_B_-resistant Gram-positive bacteria addresses a significant therapeutic gap in ophthalmology, since ocular MRSA isolates carrying *erm* genes are commonly identified [65,66]. In a collection of 50 genomically characterized ocular MRSA isolates, including 25 harboring *erm* genes, iboxamycin and cresomycin retained potent in vitro activity across the major ocular MRSA clonal complexes CC5 and CC8 [70,179]. Iboxamycin showed MIC_50_ and MIC_90_ values of 0.06 and 2 µg/mL, respectively; cresomycin showed MIC_50_ and MIC_90_ of 0.06 and 0.5 µg/mL [179]. In *erm*-positive isolates, the MIC_90_ of clindamycin was higher than 16 µg/mL, compared with 2 µg/mL for iboxamycin and 0.5 µg/mL for cresomycin [179].

### 5.7. Phages and Phage-Derived Endolysins

Bacteriophages are viruses that selectively infect and lyse target bacteria through species- or strain-specific receptor binding, followed by intracellular replication and host cell lysis. Phage-derived endolysins are recombinant lytic enzymes that cleave the bacterial cell-wall peptidoglycan directly, without requiring intracellular replication. Phage therapies are considered alternative or complementary strategies to conventional antibiotics, with the advantage of preserved activity against MDR Gram-positive isolates. Phages have a long history of clinical use in Eastern Europe and have more recently garnered interest in the U.S. and Western Europe for severe MDR bacterial infections [191].

To assess bacteriophages as potential topical ophthalmic therapies, we evaluated the in vitro antimicrobial activity of a set of anti-*S. aureus* phages against a collection of U.S. ocular MRSA clinical isolates. We showed that 90% of isolates were susceptible to at least one phage. Phages V1SA19, V1SA20, and V1SA22 had the broadest spectrum of activity across the epidemic CC5 and CC8 lineages [192]. In a mouse model of *S. aureus* endophthalmitis, a single intravitreal injection of the chimeric staphylolytic endolysin Ply187 killed intraocular bacteria and reduced the inflammatory response [182]. To date, only a limited number of studies have evaluated phage therapy in patients with ocular infections, yet clinical resolution and culture negativity were achieved in a patient with recurrent VISA/VRSA keratitis [118].

Beyond staphylococci, anti-enterococcal phages have shown similar preclinical efficacy in postoperative endophthalmitis models [193,194,195,196]. Kishimoto et al. first demonstrated that intravitreal bacteriophage therapy reduced bacterial burden, inflammatory cell infiltration, and retinal damage in a mouse model of endophthalmitis caused by both vancomycin-sensitive and vancomycin-resistant *E. faecalis* [193]. They subsequently identified three *Herelleviridae* phages (phiEF7H, phiEF14H1, and phiEF19G) with broader lytic spectra than the earlier phage phiEF24C; these phages lysed all 14 tested endophthalmitis-derived *E. faecalis* isolates and all 4 tested vancomycin-resistant isolates, and each reduced intraocular bacterial burden together with neutrophilic inflammation after intravitreal administration in mice [196]. In a rabbit cataract-surgery model, intracameral bacteriophage administration for postoperative prophylaxis suppressed postoperative *E. faecalis* endophthalmitis [195]. They also demonstrated that bacteriophages degraded *E. faecalis* biofilms on acrylic intraocular lenses and reduced viable adherent bacteria, with direct implications for device-associated infection [194].

Phage endolysins are also active against other Gram-positive ocular pathogens [197,198]. Silva et al. showed targeted killing of ocular *S. pneumoniae* isolates with the phage endolysin MSlys [197]. In fulminant *Bacillus cereus* eye infection, Mursalin et al. demonstrated that the phage lysin PlyB produced rapid bactericidal activity in vitro and therapeutic efficacy in experimental endophthalmitis and keratitis, with preservation of ocular tissues in both models [198].

Phages and phage-derived lysins thus represent a promising alternative therapy for Gram-positive ocular infection, with preclinical activity demonstrated against MDR isolates [182,192,194,198]. However, translation of phage-based therapy to clinical ophthalmology remains challenged by the limited availability of standardized therapeutic protocols. Phage activity needs to be verified for each clinical isolate by phage susceptibility testing prior to administration, a step that lengthens the time to treatment and currently relies on methods not yet harmonized across centers [199]. Therapeutic deployment on a large scale also requires Good Manufacturing Practice production, with documented amplification, purification, sterility, and lot-by-lot stability [200]. No harmonized regulatory framework currently exists for phage products, and most clinical-grade preparations are produced under hospital exemption or compassionate-use pathways [201]. For ophthalmic applications, these constraints translate into three operational requirements: a curated ocular phage bank covering the main bacteria causing eye infections, especially epidemic CC5 and CC8 MRSA lineages, rapid phage susceptibility testing on clinical isolates, and ophthalmic-grade formulations that preserve phage viability and limit intraocular inflammation [183]. Recent ophthalmic drug-delivery work has shown that engineered bacteriophage eye-drop formulations can be developed for multidrug-resistant *P. aeruginosa* keratitis [183].

### 5.8. Additional Antibiotics and Adjunctive Strategies

Ceftaroline is a fifth-generation cephalosporin with anti-MRSA activity mediated by high-affinity binding to PBP2a. Endogenous endophthalmitis is a recognized, sight-threatening complication of MRSA bacteremia that requires prompt systemic and intravitreal antimicrobial therapy [202]. In this setting, a case report described successful treatment of MRSA endogenous endophthalmitis with systemic ceftaroline used in place of vancomycin, supporting its consideration as second-line systemic therapy when ocular involvement complicates invasive MRSA disease [203].

Delafloxacin is a fourth-generation anionic fluoroquinolone that retains activity at acidic pH, a property relevant to ophthalmology because infected and inflamed ocular tissues become acidic from lactate produced by neutrophils and bacteria [204,205]. In a study of staphylococcal vitreous isolates from clinically diagnosed endophthalmitis (45 *S. aureus* and *S. epidermidis* isolates, including MRSA and methicillin-resistant *S. epidermidis*), delafloxacin showed a lower MIC_90_ (1.0 µg/mL) and a lower resistance rate (12%) than levofloxacin (8.0 µg/mL; 61%) or moxifloxacin (8.0 µg/mL; 50%) [205]. Formulation studies have also shown that delafloxacin can be incorporated into poly(lactic-co-glycolic acid) (PLGA) nanoparticles for potential topical ocular delivery [206].

Adjunctive non-antibiotic approaches do not substitute for antibacterial therapy but modify the local environment [207,208,209,210,211]. Low-concentration povidone-iodine has shown antibacterial activity in a prospective clinical pilot study of infectious keratitis [208] and in an in vivo mouse keratitis model [209]. Photoactivated Chromophore for Keratitis–Corneal Cross-Linking (PACK-CXL) has been evaluated as adjunctive therapy for bacterial keratitis, but the current Cochrane review found low-certainty evidence and clinical heterogeneity [210]. Nanoparticle systems are attractive as enabling delivery technologies for ocular anti-infectives; however, such formulations remain at the preclinical stage, with no marketed ophthalmic antibacterial nanoparticle product to date [187,188,206,211]. That promise must be balanced against material-specific ocular toxicity, which varies with composition, size, surface properties, dose, and exposure conditions [207,211].

### 5.9. Pediatric Considerations

Treatment of severe Gram-positive ocular infections in children presents additional challenges.

Vancomycin is used to treat pediatric MRSA orbital cellulitis; however, intravenous vancomycin dosing in children requires age-stratified pharmacokinetic targets that diverge from those in adults. The 2020 consensus guideline of the American Society of Health-System Pharmacists, the Infectious Diseases Society of America, the Pediatric Infectious Diseases Society, and the Society of Infectious Diseases Pharmacists recommends an area-under-the-curve (AUC) over 24 h to minimum inhibitory concentration ratio (AUC_24_/MIC) of 400 (up to 600, assuming MIC = 1 mg/L) for serious MRSA infections in children aged 3 months and older, replacing the previous target of 15–20 µg/mL because of dose-dependent nephrotoxicity [32]. Recommended empirical regimens are 15 mg/kg every 6 h in children, 15 mg/kg every 8 h in neonates of postmenstrual age 30–36 weeks, and 10–15 mg/kg every 12 h in preterm neonates younger than 30 weeks postmenstrual age, with therapeutic drug monitoring [32,33]. Volume of distribution and clearance significantly differ across neonates, which justifies additional population-pharmacokinetic-guided initial dosing in this population [212].

In pediatric infectious keratitis and endophthalmitis, age- and cooperation-dependent limitations on diagnostic sampling, the smaller volume of distribution of the pediatric eye, and the limited availability of pediatric pharmacokinetic data in ophthalmology all complicate empirical therapy [213]. Fortified topical antibiotics remain the principal delivery route for keratitis, but pediatric tolerability data for vancomycin, cefazolin, and the vancomycin-alternative candidates discussed in Section 5 are scarce. For *S. pneumoniae*, ocular macrolide resistance rates parallel those observed in non-invasive pediatric pneumococcal disease, where penicillin and macrolide resistance approach 40% in several U.S. and European surveillance studies [79]. These considerations justify cautious extrapolation of adult dosing and call for prospective pediatric ophthalmic pharmacokinetic and safety studies of emerging drugs.

## 6. Conclusions

The emergence of multidrug-resistant Gram-positive ocular pathogens and resistance to first-line topical therapies highlights the need for new treatments for sight-threatening infections. Among the vancomycin-alternative candidates reviewed, linezolid currently has the best-characterized ophthalmic profile, combining documented intraocular penetration after systemic administration, topical use in resistant keratitis cases, and intravitreal case reports. Daptomycin has the most substantial preclinical evidence for intravitreal use but requires prospective human ocular studies before broader clinical deployment. Lipoglycopeptides, synthetic retinoids, iboxamycin and cresomycin, tedizolid, delafloxacin, and bacteriophage-based approaches broaden the future therapeutic landscape but still depend on ophthalmic formulation development, ocular pharmacokinetic characterization, and in vivo efficacy validation. Integration of genomic surveillance and rapid pathogen-resistance characterization into ocular microbiology workflows will increasingly inform empirical choices and the design of next-generation therapies. A coordinated effort spanning ophthalmologic, microbiologic, and translational research is needed to preserve visual function in the antibiotic-resistance era.

## Figures and Tables

**Table 1:** Intravenous antibiotic therapy recommendations for patients presenting with orbital cellulitis.

Antibiotic	Adult Dose	Pediatric Dose
Vancomycin	15–20 mg/kg every 8–12 h (target AUC_24_ 400–600 mg·h/L) ^a^	Children ≥ 3 mo: 15 mg/kg every 6 h (target AUC_24_ 400–600 mg·h/L) ^a^; neonates PMA 30–36 wk: 15 mg/kg every 8 h; preterm < 30 wk PMA: 10–15 mg/kg every 12 h [32,33]
**plus one of the following drugs**		
Ceftriaxone	2 g per day	50–75 mg/kg/day (max 2 g/day)
Cefotaxime	2 g every 4 h	150–200 mg/kg/day divided every 6–8 h (max 12 g/day)
Ampicillin–sulbactam	3 g every 6 h	Children ≥1 year and <40 kg: 300 mg/kg/day of ampicillin–sulbactam total (ampicillin 200 mg/kg/day plus sulbactam 100 mg/kg/day), divided every 6 h; children ≥40 kg: adult dosing; maximum combined product 12 g/day.
Piperacillin–tazobactam	4.5 g every 6 h	100 mg/kg every 8 h (piperacillin component; max 4 g/dose)
	For anaerobic coverage	
Metronidazole *	500 mg IV or orally every 8 h	30 mg/kg/day divided every 6–8 h (max 4 g/day)

* Should be added to include coverage for anaerobes. PMA, postmenstrual age; AUC_24_, area under the concentration-time curve over 24 h. ^a^ Minimum inhibitory concentration (MIC) assumed 1 mg/L. Note: Selection and dosing should follow local institutional protocols and be adjusted for age, weight, renal function, allergy history, infection severity, microbiological findings, and therapeutic drug monitoring when necessary.

**Table 2:** Ophthalmic drug therapies for acute bacterial conjunctivitis.

**Aminoglycosides**	
Gentamicin 0.3%	1–2 drops 4×/day
Tobramycin 0.3%	1–2 drops 3×/day
**Fluoroquinolones**	
Besifloxacin 0.6%	1 drop 3×/day
Ciprofloxacin 0.3%	1–2 drops 4×/day
Gatifloxacin 0.3%	1 drop 3×/day
Levofloxacin 0.5%	1–2 drops 4×/day
Moxifloxacin 0.5%	1 drop 3×/day
Ofloxacin 0.3%	1–2 drops 4×/day
**Macrolides**	
Azithromycin 1%	2×/day for 2 days; then 1 drop daily for 5 days
Erythromycin 0.5%	4×/day
**Combination drops**	
Trimethoprim/polymyxin B 0.1%	1 or 2 drops 4×/day

Adapted from Azari et al. 2020 [36] and Conjunctivitis Preferred Practice Pattern AAO 2024 [35]. In *N. gonorrhoeae* conjunctivitis, systemic therapy is necessary: a single intramuscular dose of ceftriaxone 1 g; because chlamydial co-infection is common, concurrent treatment for *C. trachomatis* with doxycycline 100 mg orally twice daily for 7 days is recommended unless chlamydial infection has been excluded [35].

**Table 3:** Recommended antibiotic therapy for bacterial keratitis by AAO [24].

Organism	Antibiotic	Topical Concentration
Organism not yet identified or multiple types of organisms	Fortified cefazolin	50 mg/mL
	**With**	
	Fortified tobramycin	9–14 mg/mL
	**Or**	
	Fluoroquinolones *	3–6 mg/mL *
Gram-positive cocci	Cefazolin	50 mg/mL
	Vancomycin	25–50 mg/mL
	Fluoroquinolones *	5–6 mg/mL *
Gram-negative rods	Tobramycin	9–14 mg/mL
	Ceftazidime	50 mg/mL
	Fluoroquinolones *	3–6 mg/mL *
Gram-negative cocci	Ceftriaxone	50 mg/mL
	Ceftazidime	50 mg/mL
	Fluoroquinolones *	3–6 mg/mL *

* Fluoroquinolone concentrations vary according to the suspected or identified organism: 5–6 mg/mL for Gram-positive cocci and 3–6 mg/mL for Gram-negative rods, Gram-negative cocci, and cases in which no organism is identified or multiple organisms are suspected.

**Table 4:** Intravitreal antibiotic doses per 0.1 mL and their clinical role in bacterial endophthalmitis.

Antibiotic	Intravitreal Dose per 0.1 mL
Amikacin	0.4 mg
Ampicillin	5 mg
Aztreonam	0.1 mg
Cefazolin	2.25 mg
Cefotaxime	0.4 mg
Ceftazidime	2.25 mg
Ceftriaxone	2 mg
Ciprofloxacin	0.1 mg
Clindamycin	1 mg
Gentamicin	200 μg
Levofloxacin	0.5–0.625 mg
Moxifloxacin	160 μg
Piperacillin/tazobactam	225 µg total (piperacillin 200 µg + tazobactam 25 µg)
Tobramycin	100–400 μg
Vancomycin	1 mg

Note: All doses are expressed per 0.1 mL. Vancomycin 1 mg and ceftazidime 2.25 mg are the standard empirical intravitreal drugs for suspected bacterial endophthalmitis; amikacin 0.4 mg is an alternative when ceftazidime cannot be used, but aminoglycosides are associated with a risk of retinal toxicity.

**Table 5:** Main phenotypes and genotypes of macrolide resistance identified in *Staphylococcus* spp. and *Streptococcus* spp.

			Phenotype of Resistance			
Species/Mechanism	Gene	Phenotype Designation	14-15-MM	16-MM	C	S_B_
***Staphylococcus*** **spp.**						
Ribosomal methylation	*erm(A)*, *erm(C)*	MLS_B_ inducible	R	S	S/R *	S
		MLS_B_ constitutive	R	R	R	R
Efflux	*msr(A)*	MS_B_	R	S	S	R
Antibiotic inactivation	*mph(C)*	M	R	S	S	S
***Streptococcus*** **spp.**						
Ribosomal methylation	*erm(B)*	MLS_B_ inducible	R	R	S/R	S
		MLS_B_ constitutive	R	R	R	R
Efflux	*mef(A)*/*msr(D)*	M	R	S	S	S

* Depending on the D-test results. MM: membered macrolides; C: clindamycin; S_B_: streptogramins B; S: susceptible; R: resistant.

**Table 6:** Emerging anti-Gram-positive therapies in ophthalmology.

Agent (Class)	Mechanism	Level of Ophthalmic Evidence ^1^	Ocular Pharmacokinetics	Reported Ophthalmic Dose	Ocular Safety Profile	Regulatory Status
**Linezolid** **(oxazolidinone)**	Blocks formation of the 50S initiation complex [157,158].	In vitro; human ocular PK; topical clinical series; IVT animal data; human IVT case report [157,159,160,161].	Best-documented adjunct systemic anti-Gram-positive option: mean vitreous 5.7 ± 2.7 μg/mL after two oral 600-mg doses; ≈ 2.98 ± 1.87 μg/mL after one oral 600-mg dose [157,159].	PO/IV 600 mg every 12 h,; topical 0.2%; IVT 200 μg/0.1 mL reported [160,161,162].	Systemic myelosuppression/neuropathy risk; topical series reported better comfort than vancomycin; exudative retinal detachment reported after IVT case (causality uncertain) [158,161,162].	FDA-approved systemic formulations only; no ophthalmic approval [158].
**Daptomycin** **(lipopeptide)**	Calcium-dependent membrane depolarization [163,164,165].	In vitro; intravitreal animal models; topical in vivo keratitis model; human systemic pharmacokinetic case; human intravitreal cases. [163,166,167,168,169,170,171].	Rabbit IVT t½ ≈ 25.7 h in normal eyes and 34.6 h in inflamed eyes; systemic IV case achieved vitreous level ≈28% of serum [166,167].	IVT 200 μg/0.1 mL used clinically; 200 μg/0.05 mL; topical 1% studied in vivo (rabbit) [163,166,168,169].	Dose-dependent cataract, ERG suppression, and photoreceptor damage at ≥375 μg in rabbits; acceptable safety around 75–200 μg [163].	FDA-approved systemic formulations only; no ophthalmic approval [164].
**Dalbavancin, oritavancin, telavancin (Lipoglycopeptides**)	Cell-wall inhibition plus lipophilic membrane anchoring; some agents show faster kill kinetics than vancomycin in vitro [172,173,174,175].	Ocular in vitro activity and corneal-cell cytotoxicity; topical in vivo murine tolerability data for telavancin; no ocular infection-efficacy or clinical data. [172,176].	No clinical ocular PK or IVT data identified.	No ophthalmic dose validated; corneal-cell work used 25 mg/mL comparator conditions [172]. Topical telavancin 25 and 50 mg/mL evaluated in a murine tolerability study [176].	Concentration- and time-dependent HCEC cytotoxicity in vitro. In mice, telavancin 25 mg/mL was better tolerated than vancomycin 25 mg/mL [172,176].	FDA-approved systemic agents only; no ophthalmic approval [173,174,175].
**CD437, CD1530 (Synthetic retinoids**)	Membrane-disruptive small molecules with anti-biofilm activity [177,178].	Ocular in vitro only.	No ocular PK data.	No ophthalmic dose validated.	Ocular safety window unknown; translation requires formulation and toxicity work [177].	Investigational only.
**Iboxamycin/Cresomycin** **(synthetic lincosamides)**	Ribosome-targeting synthetic lincosamides optimized to retain activity despite Erm/Cfr-mediated target modification [179,180,181].	Ocular MRSA in vitro [179].	No ocular PK data.	No ophthalmic dose validated.	No ocular safety dataset yet.	Investigational only.
**Phages/phage endolysins**	Bacterial lysis [182,183].	In vitro/in vivo; case reports [182,183].	No standardized PK framework; exposure depends on formulation, ocular compartment, and pathogen matching [182,183].	No standard human dose.	Preclinical ocular tolerance is encouraging, but manufacturing, immunogenicity, and pathogen-matching remain barriers.	No approved ophthalmic phage product identified.

Abbreviations: MRSA, methicillin-resistant *Staphylococcus aureus*; PK, pharmacokinetics; IVT, intravitreal; PO, oral; IV, intravenous; HCEC, human corneal epithelial cells. ^1^ Level of ophthalmic evidence: graded from in vitro microbiology, through ocular animal models (keratitis/endophthalmitis), to human ocular case reports or clinical series. “Regulatory status” refers to currently available systemic labels versus absence of ophthalmic approval in the cited sources.

## Data Availability

No new data were created or analyzed in this study. Data sharing is not applicable to this article.
